# Individualised dosing strategy for high-dose carboplatin in patients with germ cell cancer

**DOI:** 10.1038/sj.bjc.6601215

**Published:** 2003-08-26

**Authors:** C Kloft, W Siegert, U Jaehde

**Affiliations:** 1 Department of Clinical Pharmacy, Insitute of Pharmacy, Freie Universitaet Berlin, Kelchstr. 31, 12169 Berlin, Germany; 2 Department of Haematology and Oncology, Charité, Campus Mitte, 10117 Berlin, Germany; 3 Department of Clinical Pharmacy, Rheinische Friedrichs-Wilhelms-Universitaet Bonn, 53121 Bonn, Germany

**Keywords:** high-dose carboplatin, dose individualisation, germ cell tumour, high-dose chemotherapy, population pharmacokinetics

## Abstract

In contrast to conventional chemotherapy, carboplatin is still dosed per unit of body surface area (BSA) in high-dose chemotherapy protocols in clinical practice. To individualise dosing, a population pharmacokinetic model for poor-risk germ cell tumour patients receiving 1500 mg m^−2^ carboplatin was developed. The typical central volume of distribution (19.9 l) and typical clearance (110 ml min^−1^) corresponded approximately to the extracellular fluid space or glomerular filtration rate, respectively. The covariate analysis identified several patient-specific factors. Carboplatin clearance was significantly related to creatinine clearance and body height, explaining 73% of the interindividual variability. Thus, an equation to predict individual clearance prior to treatment was developed (CL=0.41 × creatinine clearance+1.05 × body height−124.4). The relative frequency of developing toxicity increased significantly with higher AUC values for different types of toxicity. In addition, overall nonhaematological toxicity correlated significantly with exposure of carboplatin, leading to the assessment of a target AUC. Based on the prediction of individual clearance and the definition of a target AUC associated with moderate toxicity, an individualised dosing equation is proposed. Retrospectively, the individualised dosing strategy would have led to a higher dose on average and a broader range to be administered, compared to empirical dosing per unit BSA in the high-dose setting.

Anticancer agents are usually dosed per unit of body surface area in clinical practice. Carboplatin, an active component in potentially curative regimens of different solid tumours, is one of the few examples that in *conventional* dosing is not dosed according to body surface area. For conventional-dose carboplatin, Egorin and Jodrell (1992) demonstrated significant correlations between platelet nadir (expressed as the relative change from baseline) and pharmacokinetics (PK), namely the area under the concentration–time curve, AUC. Based on the correlations found, they defined a target AUC associated with a platelet nadir that was considered to be tolerable. Using this target AUC concept, they suggested two different dosing formulas depending on whether patients had been pretreated with chemotherapy or not. Calvert *et al* (1989) presented a dosing formula to individualise the dose based on the patient's renal function, pretreatment with cytotoxic agents and chemotherapy protocol (monotherapy or combination therapy). This dosing formula for conventional-dosed carboplatin has been approved by regulatory agencies, for example, FDA in 1997. In prospective clinical trials, it was demonstrated that individualisation resulted in a more precise prediction of the effect of carboplatin in terms of myelosuppression, especially thrombocytopenia–the dose-limiting toxicity (Jodrell *et al*, 1992; Gore *et al*, 1998). In high-dose chemotherapy (HDCT) followed by autologous stem cell rescue (ASCR), however, myelosuppression is no longer the dose-limiting toxicity. Other types of toxicity will have to be considered instead (Wright *et al*, 1995; Huitema *et al*, 2002; Kloft *et al*, 2002), and the therapeutic index for the patients has to be newly defined. Although the Calvert dosing formula has been applied to high-dose treatment (Motzer *et al*, 2000), so far pharmacokinetic/pharmacodynamic analysis is rare (Huitema *et al*, 2002). In germ cell cancer patients, high-dose chemotherapy is a highly valuable option for poor-risk patients (Siegert *et al*, 1998; Rodenhuis *et al*, 1999). We therefore performed a study in this patient population in order to investigate comprehensively the population pharmacokinetics and pharmacokinetics-toxicity relationships of high-dose carboplatin, resulting in an individualised dosing recommendation. In addition, a comparison with existing strategies was carried out.

## PATIENTS AND METHODS

### Patient selection/enrolment

Eligibility for HDCT with ASCR required a histologically or tumour marker proven germ cell tumour, multiple relapse and/or refraction to prior chemotherapy, a Karnofsky index ⩾60% and an age ⩾18 years. Patients were not recruited if one or more organs were more than slightly impaired (kidney, e.g., 1.3-fold of reference value or creatinine clearance <70 ml min^−1^, liver, lung, heart and bone marrow), or the bone marrow was infiltrated by the tumour. Prior to HDCT, all patients had received cisplatin-based conventional dose chemotherapy. The last cycle consisted of either cisplatin, etoposide and ifosfamide (PEI) or cisplatin, ifosfamide and paclitaxel (TIP).

Prior to start of the study, the protocol was approved by the Ethics Committee of the University hospital and all patients gave written informed consent.

### Treatment protocol

The HDCT protocol for the treatment of germ cell tumour patients consisted of 500 mg m^−2^ day^−1^ carboplatin as a 1-h i.v. infusion for three consecutive days: days −6 to −4 (i.e., in total 1500 mg m^−2^). If renal function was mildly impaired prior to HD treatment, that is, a creatinine clearance of 70–85 ml min^−1^, the carboplatin dose was reduced by 20%. Carboplatin administration was followed by etoposide (600, or 450 mg m^−2^ day^−1^ in renal impairment, as a 1-h i.v. infusion for 4 days: days −6 to −3) and a third cytotoxic agent that was either ifosfamide (2500 mg m^−2^ day^−1^ as a 22-h i.v. infusion for 4 days: days −6 to −3, i.e, CEI regimen) or thiotepa (250 or 150 mg m^−2^ day^−1^ as 1 h- i.v. infusion for 3 days; days –6 to –4, i.e., CET regimen). At 3 days after the last etoposide administration on day 0, an ASCR was performed. Stem cells were either recruited from bone marrow or peripheral blood after stimulation with G-CSF. In addition, patients received supportive drug treatment, for example, antiemetic, uroprotective (with MESNA, only in the CEI regimen), anti-infective and hyperuricemia prophylaxis.

A total of 29 patients were treated according to the protocol. The summary statistics of the patients' characteristics are listed in Table 1

**Table 1:** Summary table of patient characteristics

**Demographics**	***n***	**x̄**	***s***	**RSD (%)**	**x̃**	***I*_80_**
Age (years)	29	34.0	8.7	25.6	32.9	20.9
Weight (kg)	29	76.7	14.5	18.9	74.0	41.2
Height (cm)	29	178.5	7.1	4.0	178.0	18.4
BSA (m^2^)	29	1.9	0.2	9.6	1.9	0.5
Creatinine conc. (mg dl^−1^)	29	0.96	0.15	15.6	0.90	0.36

x̄=arithmetic mean; s=standard deviation; RSD=relative standard deviation; x̃=median; *I*_80_=difference between the 9th and 1st decile, that is, the range that covers 80% of sample distribution.

. Typical of this malignancy, the median age was 33 years (range 20–54 years). In all, 23 patients received the full HDCT regimen (12 CEI regimen, 11 CET regimen) and six the reduced regimen due to a mild renal impairment prior to HDCT (all CET regimen). As ASCR, in 10 patients bone marrow was retransplanted while in 19 patients peripheral blood stem cells were retransfused.

### Sample collection and bioanalysis

For the determination of PK parameters, serial blood samples were drawn from the beginning of the HDCT until the day of the ASCR at the following time points: prior to and at 0, 7 and 23 h after the end of the first and second carboplatin infusion, as well as 10, 20 and 30 min after the start and 0, 1.5, 4, 7, 11, 23, 27, 35 and 96 h after the end of the third infusion (group 1). After the first 10 patients, an optimised schedule was applied, that is, prior to and at 0, 1.5, 2.5, 4, 7, 11, 21 h after the end of the first infusion, 0 and 23 h after the end of the second and 0, 1.5, 4, 7, 11, 23, 35, 44, 56, 72 and 96 h after the last infusion (group 2). For PK parameter estimation, the actual time points of sample collection were considered.

Sample pretreatment at the hospital and the bioanalytical methods for the determination of platinum have been described in detail previously (Kloft *et al*, 1999). In short, blood samples were centrifuged and plasma was further centrifuged either in Centrisart™ (Sartorius, Goettingen, Germany) or in Centrifree™ tubes (Millipore, Eschborn, Germany) to prepare ultrafiltered plasma aliquots where the platinum species not bound to macromolecules could be analysed. All aliquots were stored at −70°C until analysis. The validated flameless atomic absorption spectrometry (FAAS) method for the determination of platinum in ultrafiltrate only required 40 *μ*l sample volume of the patient. The calibration curve of the assay was linear from 0.021 to 32 000 *μ*g Pt ml^−1^ with coefficients of variation of 1.2–7.3 and 2.9–8.6% for within-day and between-day precision, respectively, across the concentration range (Kloft *et al*, 1999).

### Population pharmacokinetic data analysis

For the determination of PK parameters of carboplatin, the measured platinum concentrations were multiplied by the molar mass ratio 1.903 (carboplatin : platinum) in order to compare them with the results of other authors. Pharmacokinetic analysis was carried out using the software P-Pharm™ version 1.4 (SIMED, Créteil Cedex, France). Ultrafiltrate concentration–time data of all patients were simultaneously evaluated in order to characterise the PK profile of the patient population with mean and variability PK parameters (basic PK model). The variability parameters included interindividual (between patient) variability, assumed to be normally distributed (*P*<0.05, Shapiro–Wilk test for normality) and modelled as additive error, and residual (within one patient) variability modelled as proportional heteroscedastic error. In addition, a covariate analysis was performed. Several patient-specific factors (covariates) were evaluated for displaying a significant influence on PK parameters. The aim was to find and to quantify the relationships between covariates and the individual predicted PK parameters. The covariates tested included age, body weight, body height, body surface area (BSA), concomitant medication of ifosfamide and various measures of kidney function. A stepwise multiple regression analysis implemented in the software P-Pharm™ was performed according to the following equation (Draper and Smith, 1966):


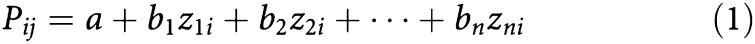


where *P_ij_* is the *i*th individual predicted PK parameter *j*, *a* the intercept, *b_n_* the coefficient for the *nth* covariate found to be significant and *z_ni_* the *i*th individual *n*th covariate. On each step, the covariate with the highest partial correlation coefficient was entered into the regression equation. At every stage of the procedure the contribution of each covariate, entered at the current step or previously, was examined. An ANOVA was performed for statistical significance, retaining or rejecting the covariates based on their partial *F* values (Draper and Smith, 1966). The procedure was finished if no covariate could either be entered or rejected from the regression equation (Draper and Smith, 1966; Gomeni *et al*, 1994). Significant covariates identified were finally included into the PK model (final PK model). Mean PK parameters and significant covariates were regarded as fixed-effects parameters and interindividual and residual variability as random-effects parameters.

For each step the explained percentage of the interindividual variability of a significant covariate was calculated according to (Draper and Smith, 1966):


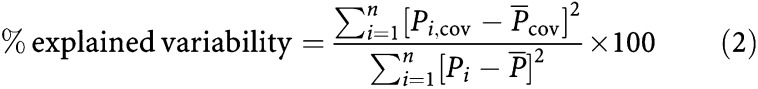


where *n* is the number of patients, *P_i_* is the individual model predicted PK parameter and *p̄* is the mean population PK parameter, that is, both according to the basic PK model. *P*_*i*, cov_ is the individual predicted PK parameter according to the covariate model (eq. 1) and *p̄*_cov_ is the respective mean value.

### Toxicity evaluation

Toxicity of various organs (heart, lung, liver, kidney, skin, ear), central and peripheral nervous system (CNS, PNS), and GI tract motility was evaluated daily according to modified WHO classification criteria (categorical scale: 0–4, where grade 0 represents no toxicity and grade 4 life-threatening toxicity (McGuire, 1979)). Apart from serum creatinine concentration, renal function was further assessed by creatinine clearance, estimated for all 29 patients (Cockcroft and Gault, 1976) and determined by a 24-h urine collection, CLCR_24 h_, *n*=26, as well as by ^51^Cr-EDTA clearance, *n*=25.

In order to examine whether there was a higher probability of a patient experiencing a certain type of toxicity if exposure of carboplatin was higher, the occurrence of toxicity was related to the area under the concentration–time profiles (AUC) of high-dose carboplatin calculated from individual PK analysis. For this purpose, groups of patients with different AUC values were formed, and for each of the groups the relative frequency of patients developing toxicity was calculated.

### Dose individualisation

Development of an individualised dosing strategy was based on the target AUC concept and individual clearance prediction.

### Target AUC

For the patient population investigated, the decision for a target AUC value was based on the relationships between AUC and toxicity, that is, the target AUC determined will then result in a certain extent of toxicity.

### Individual CL prediction

For conventional carboplatin, there are various strategies for predicting individual clearance. The predictive performance of the two most important strategies, proposed by Calvert *et al* (1989) and Chatelut *et al* (1995), for predicting carboplatin CL of the HDCT patients studied was evaluated using the mean prediction error (MPE) as a measure of bias and the root mean squared prediction error (RMSE) as a measure of precision according to (Sheiner and Beal, 1981)


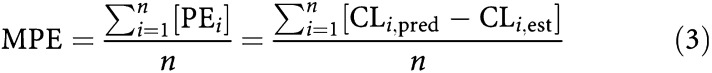



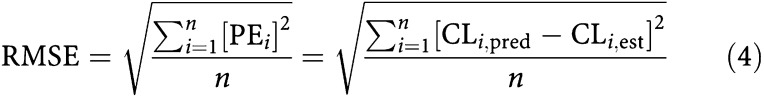


where *n* is the number of patients *i* and PE is the prediction error of each pair, that is, the difference between the predicted and the estimated (individual model predicted found in this study) value, CL_*i*, pred_−CL_*i*, est_.

The Calvert and Chatelut equations for male subjects are as follows:


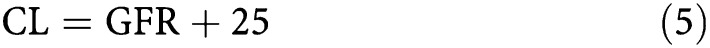


where the GFR is the glomerular filtration rate (ml min^−1^) and


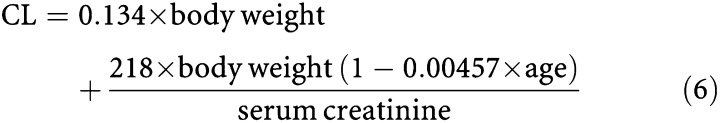


Here, serum creatinine concentration has to be in *μ*M. Originally, Calvert *et al* based the GFR determination on the clearance of ^51^Cr-EDTA. As other authors have suggested using creatinine clearance for GFR instead for practical purposes, creatinine clearance based on the Cockcroft–Gault equation (CLCR_CG_) and on the 24-h urine collection (CLCR_24 h_) were investigated as well.

### Statistical methods

If not stated otherwise, all data are presented as mean±s.d. (*s*). The data were analysed using the software programs STATGRAPHICS *Plus*™ version 1.0 (Manugistics, Rockville, MD, USA), S-STAT Package™ version 4.0 (SIMED, Créteil Cedex, France) and SPSS™ version 7.5 (SPSS Inc., Chicago, USA). A *P*-value of ⩽0.05 was considered statistically significant.

## RESULTS

All 29 patients entering the study were evaluated and are reported in the following.

### Population PK

**Figure 1:**
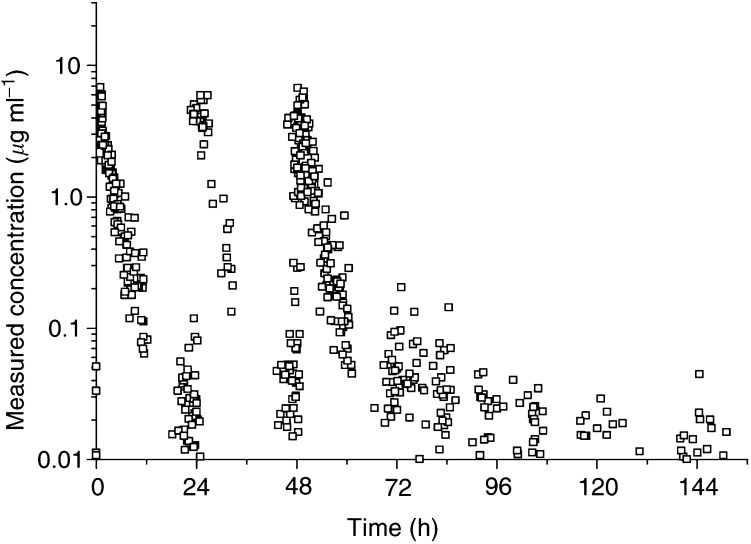
Time course of all measured concentrations in ultrafiltered plasma after the three carboplatin infusions (*n*=557).

In total, 557 ultrafiltrate concentration–time data were available for the population PK analysis. The number of observations per patient ranged from 13 to 23. Figure 1

shows the concentration–time profile of all data sets. An open two-compartment model with zero-order input and first-order elimination from the central compartment best described the data. The basic PK parameters of clearance (CL), central volume of distribution (*V*_c_) and the transfer rate constants (*k*_12_, *k*_21_) are listed in Table 2

**Table 2:** Population pharmacokinetic parameters of the basic PK (without covariates) and final PK model (with covariates)

	**Basic model**		**Final model (according to Eq. 1)**			
**Parameter**	**Mean**	**Variance**	**Mean**	**Variance**	**Intercept**	**Coefficient(s)**
CL	110.0	493	110.5^a^	171	−124.4	CLCR_24 h_: 0.408
(ml min^−1^)						
						Height: 1.05
*V*_c_	19.8	3.6	19.9^a^	2.5	12.9	Weight: 0.091
(l)						
*k*_12_	0.042	1.3 × 10^−4^	0.042^a^	6.6 × 10^−5^	−2.6 × 10^−2^	Weight: −4.7 × 10^−4^
(*h*^−1^)						Height: 7.5 × 10^−4^
						Age: −6.4 × 10^−4^
						CLCR_24h_: −8.1 × 10^−5^

*k*_21_	0.024	2.1 × 10^−5^	0.023	2.1 × 10^−5^	—	—
(h^−1^)						

a For a patient with average values of the covariates.

(left part). The population PK was characterised by a mean CL of 110 ml min^−1^ that corresponded approximately to the glomerular filtration rate of the kidney. Regarding interindividual variability, the error variance was lowest for *V*_c_ and highest for CL and *k*_12_. The residual variability was 6.8%. Individual Bayesian PK parameters, that is, individual predicted PK parameters, were generated based on the population parameters of the model and on the individual concentration–time data. The covariate analysis investigating the relationships between the patient-specific factors and individual predicted PK parameters selected several covariates as influential on CL, *V*_c_ and *k*_12_ but not on *k*_21_ (Table 3

**Table 3:** Selected covariates with influence on the pharmacokinetic parameters

**Parameter**	**Covariate**	**Explained interindividual variability (%)**
CL	CLCR_24 h_	63
	CLCR_24 h_ and body height	73
*V*_c_	Body weight	63
*k*_12_	Body weight	23
	Body weight and body height	47
	Body weight, body height and age	63
	Body weight, body height, age and CLCR_24h_	68
*k*_21_	None	0

**Figure 2:**
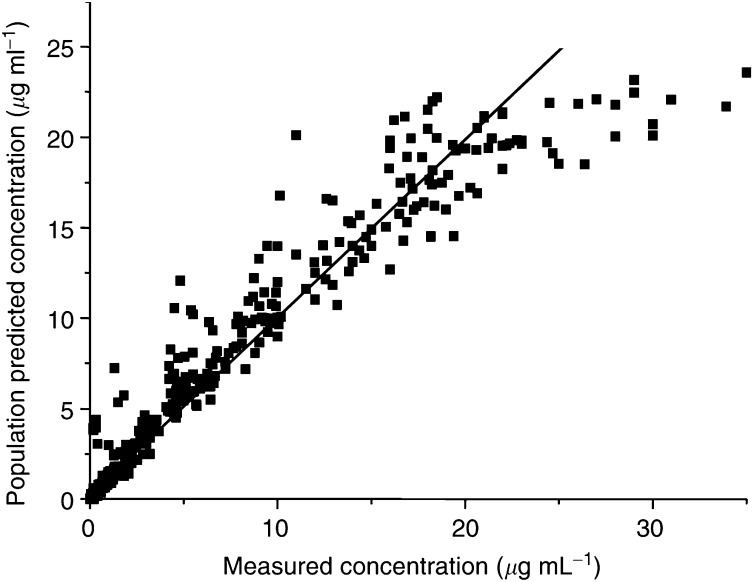
Correlation between measured and model-predicted concentrations in ultrafiltered plasma (*n*=557).

CLCR_24 h_=creatinine clearance determined by the 24-h urine collection method.

). Creatinine clearance determined by the 24-h urine collection method (CLCR_24 h_) explained almost two-thirds of the interindividual variability of CL. The inclusion of body height, increased explained variability to 73%. If weight was included in the analysis instead of height, only renal function remained as a significant covariate for CL. Renal function determined by ^51^Cr-EDTA clearance was less explanatory (only 29%) than CLCR_24 h_, which was closely monitored in clinical routine in the HDCT patients. The covariates selected were then included in the PK model in order to establish the final relationships between the covariates and the PK parameters. The final mean and variability PK parameters are summarised in Table 2 (right part). The unexplained interindividual variability was most reduced for CL, that is, more than 40%. In Figure 2

, the population predicted concentrations, based on the final model, are plotted against the measured concentrations. The values below 24 *μ*g ml^−1^ were relatively symmetrically distributed around the line of identity, indicating that the PK model adequately described the PK profile of HD carboplatin. Although in the high concentration range an underprediction can be observed suggesting, for example a three-compartment model, a two-compartment model resulted in a better fit to the data. Closer examination of these values, obtained from a few individuals directly at the end of the infusions, in an individual PK analysis revealed, for some patients, that a three-compartment model gave better results with an additional extremely short ‘distribution’ phase just after the end of the infusions.

### Correlation of PK and toxicity

All patients experienced various types of toxicity of WHO grades ⩾1. If patients already showed a grade ⩾1 toxicity prior to therapy, only the difference was considered. All patients developed grade 4 haematotoxicity, but haematological recovery was successful in all. Other types of toxicity did not occur in all patients.

**Figure 3:**
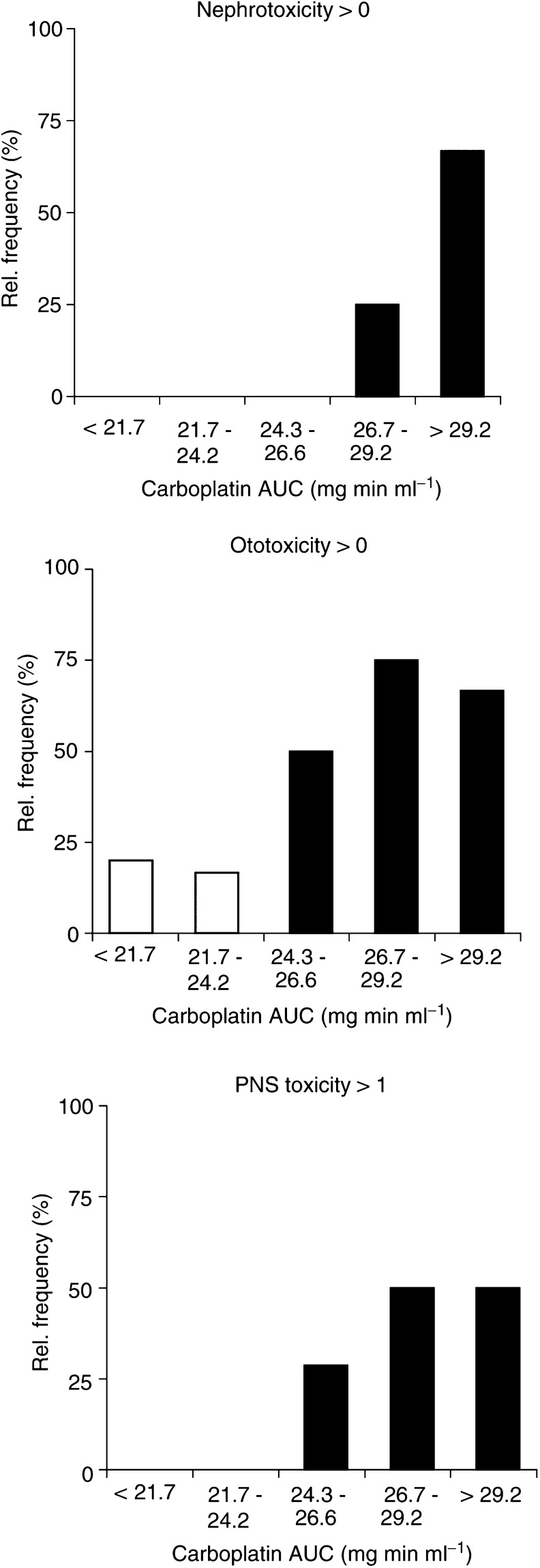
Relative frequency of five groups with patients of certain carboplatin AUC values to develop nephrotoxicity, ototoxicity and PNS toxicity (*n*=5,6,7,8,3, respectively, starting with the group of lowest AUC values).

As explorative examination of a relation of AUC values, ranging considerably from 16.4 to 42.0 mg min ml^−1^ with approximately 75% of the values between 21.7 and 29.2 mg min ml^−1^, and the occurrence of toxicity, the relative frequency of five groups of patients with different AUC values (three groups within the range covering the 75% sample distribution as well as one group with lower and one with higher values) is illustrated in Figure 3

. For PNS toxicity, the analysis was performed with grades > 1 since almost all patients developed at least grade 1 PNS toxicity. For nephrotoxicity, ototoxicity and PNS toxicity, the relative frequency of developing toxicity was increased with higher exposure, most pronounced for renal toxicity. No patient in the lower three AUC groups experienced renal toxicity, whereas 25% of the patients with AUC values between 26.7 −29.2 mg min ml^−1^ and 75% with AUC values above 29.2 mg min ml^−1^ developed renal toxicity. Due to the small number of patients and the explorative nature of the examination, no further statistical analysis was performed.

**Figure 4:**
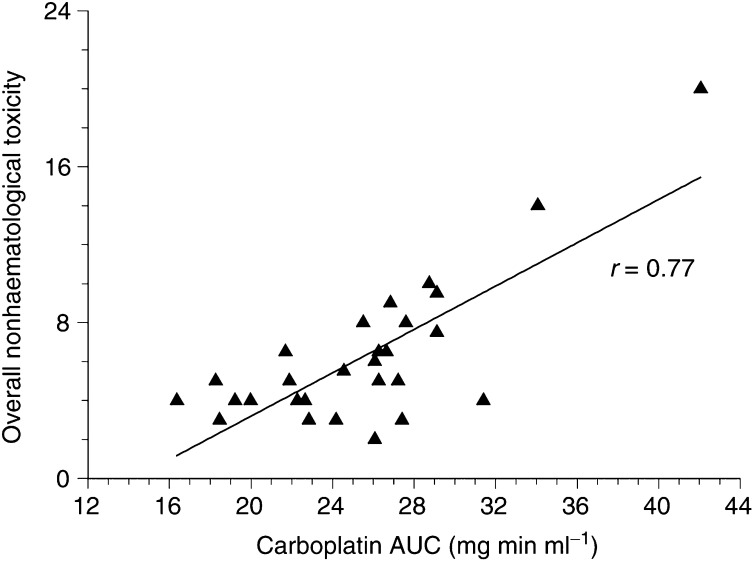
Overall nonhaematological toxicity in relation to AUC of carboplatin (*n*=29).

In addition, the maximum therapy-associated toxicity grades were evaluated in relation to exposure. As a measure of the extent of overall nonhaematological toxicity for each patient, all individual toxicity scores were added. This individual sum of the overall nonhaematological toxicity was related to the AUC of carboplatin. As illustrated in Figure 4

, the Pearson correlation coefficient between exposure and overall nonhaematological toxicity was statistically significantly different from zero (*P*<0.01).

### Dose individualisation

#### Target AUC

The patient population displayed a large difference (2.6-fold) in AUC values. If almost no toxicity is to be accepted, a low(er) target AUC of approximately 24 mg min ml^−1^ might be considered. Based on the significant correlation between AUC and the overall nonhaematological toxicity, we suggest a target AUC in the order of 26–27 mg min ml^−1^. This target AUC, that is exposure of carboplatin, is associated with moderate occurrence of toxic effects. A higher AUC would be associated with a higher risk of (unacceptable) toxicity. The patient with the highest AUC value of 42.0 mg min ml^−1^ suffered from multiple grade 3–4 toxicities, which further strengthens the demands of a tailored dose according to a target AUC.

#### Individual CL prediction

The results of predictive performance of the four established strategies for conventional carboplatin are presented in Table 4

**Table 4:** Predictive performance of different methods to predict individual clearance of patients treated with high-dose carboplatin

**Method**	**Description**	**Precision (RMSE in ml min^−1^)**	**Bias (MPE in ml min^−1^)**
1	Calvert (eq. 5): GFR as CLCR_CG_	38	+32
2	Calvert (eq. 5): GFR as CLCR_24 h_	37	+27
3	Calvert (eq. 5): GFR as CL_51_Cr-EDTA__	30	+1.3
4	Chatelut (eq. 6)	72	+67
(Eq. 7)	Based on CLCR_24 h_ and body height	16	−4.1

CLCR_CG_=creatinine clearance based on the Cockcroft–Gault equation; CLCR_24 h_=creatinine clearance determined by the 24-h urine collection method

CL_51_Cr-EDTA__=^51^Cr-EDTA clearance; RSME=root mean squared prediction error; MPE=mean prediction error.

. All methods for predicting the CL of the patients in this study exhibited relatively poor precision (RMSE at least 30 ml min^−1^). Methods 1, 2 and 4 resulted in pronounced biased predictions, that is, the CL values were largely overestimated. The ‘original’ Calvert equation (method 3) yielded unbiased predictions on average, that is there was no systematic error, but the individual predictions deviated from the respective individual estimates between −49 and 101 ml min^−1^. The Chatelut equation showed the highest deviations regarding accuracy and precision.

Since all methods for conventional carboplatin dosing did not predict CL in high-dose treatment satisfactorily, a new prediction method for individual CL is proposed. The equation is based on the results of the covariate relationships of the final PK model:


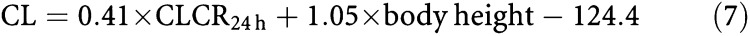


CLCR_24 h_ (in ml min^−1^) and body height (in centimeter) were the only patient-specific factors influencing individual clearance (ml min^−1^) resulting in a good predictive performance (MPE −4.1 ml min^−1^; RMSE 16 ml min^−1^). One has to keep in mind that these values are based on the developed equation.

### Individual dosing calculation

Based on the relationship between the PK parameters CL and AUC, and dose as *D*=CL × AUC, an individualised carboplatin dosing equation was derived from the equation of the individual predicted clearance (eq. 7) and a target AUC associated with a moderate toxicity of 26.6 mg min ml^−1^, as an example for clinical practice:


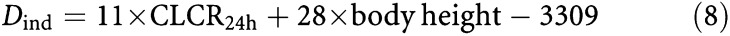


where *D*_ind_ is the individual carboplatin dose in milligram. Since it was our aim to propose a simple dosing guideline calculation for clinical practice, in the final individual dosing equation figures have been rounded off. Evaluation of the rounding effect showed only marginal differences. If one is determining patient's height, creatinine clearance (24-h collection urine) and a target AUC (here 26.6 mg min ml^−1^), the individualised carboplatin dose can be calculated prior to high-dose treatment. The equation developed is only applicable for patients with normal or slightly impaired renal function, that is, for patients usually receiving carboplatin.

Retrospectively, the proposed equation was applied to the HDCT patient population investigated and compared to the empirical BSA-based carboplatin doses administered in the study. The empirical dosing, multiplying the protocol dose of 1500 mg m^−2^ with the BSA of the patient, resulted in an average absolute carboplatin dose of 2757 mg (range 1950–3300 mg, Table 5

**Table 5:** Comparison of different dosing strategies for the patient population investigated: empirical *vs* individualised dosing

	**Empirical dosing: Dosing based on 1500 mg m^−2^ (BSA)**	**Individualised dosing: Dosing based on the proposed equation (eq. 8)**
*x̄*	2757 mg	2859 mg
Minimal dose	1950 mg	1899 mg
Maximum dose	3300 mg	4069 mg

). The corresponding individualised carboplatin dose taking the individual creatinine clearance and height into consideration was approximately 100 mg higher. Thus, on average, a higher dose could have been administered. The minimum and maximum individualised doses were lower or higher than the empirical ones, respectively. Comparing the empirical and individualised dosing for each patient separately, in two-thirds of the patients the doses differed by more than 5% up to 35%.

## DISCUSSION

Dosing in cancer chemotherapy is traditionally based on BSA and, even nowadays, most new drugs are approved in doses per unit BSA. In 1990, Grochow *et al* (1990) have highly questioned the rationale of this dosing strategy and thereby stimulated research in this field. A prerequisite for improved dosing is the study of the PK and their relationships to the effects. For conventional carboplatin dosing, many authors have thoroughly investigated the PK in different patient populations (adults and paediatrics; Shea *et al*, 1989; Newell *et al*, 1993) and also a population PK analysis was performed (Chatelut *et al*, 1995). For individualising carboplatin in HDCT, we have developed a population PK model to characterise the population and the variability. Analogous to individual data analysis (Shea *et al*, 1989; Kloft *et al*, 2002; Newell *et al*, 1987; van Warmerdam *et al*, 1996b), the population PK model did not include any nonlinear process for high-dose carboplatin. Comparable to conventional dosing, the central volume of distribution appeared to correlate approximately with the extracellular fluid space, and CL corresponded approximately to the GFR of kidneys (Chatelut *et al*, 1995). Regarding interindividual variability, we also found it highest for CL, that is, AUC, and *k*_12_ (Chatelut *et al*, 1995). To reduce interindividual variability, we performed an analysis to identify significant covariates and assess their magnitude of influence on interindividual variability. CLCR_24 h_ was the best predictor for carboplatin CL. Others have proposed ^51^Cr-EDTA or serum creatinine concentration as a marker instead (Chatelut *et al*, 1995; van Warmerdam *et al*, 1996a; Wright *et al*, 2001). If we excluded CLCR_24 h_ we also found these covariates to be significant; both markers were however less predictive. Although collection of 24-h urine is generally considered to be imprecise (van Warmerdam *et al*, 1996a), we feel that for HDCT or other patient populations where an accurate determination of fluid balance is daily routine, the urine collection method should be preferred.

Interestingly, BSA was not identified as significantly influencing CL of the population investigated. The formula to determine BSA and the derived nomogram most commonly used in clinical practice were developed at the beginning of the last century including only nine nonobese adults (DuBois and DuBois, 1916). Later examinations have shown either an over- or underprediction of the actual determined BSA (Gehan and George, 1970; Mitchell *et al*, 1971). Moreover, the appropriateness using BSA in dose calculations has been questioned for many anticancer agents (Reilly and Workman, 1994; Baker *et al*, 1995; Ratain, 1998). Except for docetaxel, where BSA has been found to be a main predictor for clearance (Bruno *et al*, 1996), recent investigations on various drugs demonstrated that BSA is poorly correlated with pharmacokinetic parameters (de Jongh *et al*, 2001; Loos *et al*, 2000; Mathijssen *et al*, 2002). Although in our study height, also a measure of body size, was identified as the second significant covariate of clearance, it accounted for only approximately 10% of the variability between patients, while weight was not selected at all. For volume of central compartment only weight, but neither height nor BSA, was a significant covariate. Thus, our data clearly suggest that BSA is not a rational factor to base high-dose carboplatin dosing upon.

In terms of PK-effect relations, we focused on exposure–toxicity relationships. Generally, carboplatin is considered to be less toxic than cisplatin concerning the effects on kidney, ear and the nervous system (Go and Adjei, 1999). In high-dose carboplatin, however, nephrotoxicity, ototoxicity and neurotoxicity also become significant as reported by others, too (Wright *et al*, 1995). Nephrotoxicity is considered to be the most frequent and dose-limiting toxicity while being reversible in the majority of patients. In our study, we found an increased probability of a patient developing nephrotoxicity, ototoxicity or PNS toxicity with increased AUC values, which was most pronounced for nephrotoxicity. Huitema *et al* (2002) also demonstrated a significant relationship between carboplatin AUC and ototoxicity. In another study with high-dose carboplatin as continuous infusion, higher steady-state concentrations were associated with a more frequent occurrence of renal impairment (Wright *et al*, 1995).

In addition to the relations of individual types of toxicity, we found a statistically significant correlation between AUC and overall nonhaematological toxicity associated with the high-dose treatment. Thus, a patient with higher carboplatin exposure is more likely to experience ‘more’ toxicity in terms of more severe and/or more different types of toxicity. Although it is not common to add toxicity grades, we think that this method is of great interest, and can be justified since the WHO classification system provides a 5-grade scale for all types of toxicity from none to life-threatening toxicity. Based on our results, a target AUC associated with acceptable toxicity (low to moderate) in the range of 24–27 mg min ml^−1^ is proposed, approximately 4–5-fold higher compared to the target values proposed for conventional-dose carboplatin (Calvert *et al*, 1989). Several groups have applied the Calvert formula to calculate carboplatin doses for patients undergoing ASCR using empirically elevated target AUC values ranging from 12–32 mg min ml^−1^ (Rodenhuis *et al*, 1999; Chatelut *et al*, 2000; Motzer *et al*, 2000). While Rodenhuis *et al* used a fixed target AUC of 20 mg min ml^−1^ leading to manageable toxicity, Motzer *et al* performed a target AUC escalation cohort study with a desired target AUC of 12–32 mg min ml^−1^ using ^99M^Tc-DTPA CL. The group reported moderate toxicity and that the measured AUC values did not reach the desired target AUC. In fact, instead of the target 32 mg min ml^−1^, the actual measured AUC values in this cohort were only 13, 22 and 23 mg min ml^−1^. Our proposed target AUC range based on PK data analysis corresponds very well to these results, and the exemplary target AUC value of 26.6 mg min ml^−1^ represents a moderate dosing strategy with regard to toxicity and thus might avoid underdosing. The two patients with the lowest carboplatin AUC in our study unfortunately experienced progressive disease directly after HDCT. If a more aggressive strategy is to be applied, the target AUC must be selected below 42 mg min ml^−1^ as toxicity associated with this AUC was too severe, leading to multiorgan dysfunction in one patient.

The second PK parameter to be controlled for dose individualisation is CL. As has been reported by others, neither the Calvert nor the Chatelut formula predicted clearance of high-dose carboplatin without bias and/or imprecision (van Warmerdam *et al*, 1996a; Motzer *et al*, 2000). Interestingly, Chatelut *et al*, 2000 has recently published an investigation where an empirical target AUC of 20 mg min ml^−1^ and individual clearance based on the formerly developed equation was used. Unlike in our study, the Chatelut formula adequately estimated carboplatin clearance. Chatelut *et al* (2000) also proposed a very versatile limited sampling strategy of two samples only, which represents an attractive approach in future studies.

Dose individualisation based on target AUC and individual CL is often associated with lowering the dose. In contrast, this strategy aims to adjust the dose to achieve a target AUC based on patient-specific factors. Compared to empirical dosing based on BSA, the proposed tailored dose represented an increase in more than 40% and a decrease in only 24% of the patients. Individualised dosing taking renal function, body height and an acceptable toxicity into account therefore results in a wider dose range than the empirical dosing. *A priori* adaptive dosage determination based on the relative contribution of identifiable patient-specific characteristics and a reasonable target AUC might therefore result in a more favourable outcome. A dosage formula based on the retrospective analysis of the PK of carboplatin in 29 patients has been developed, which should be further evaluated in patients with different types of tumour and chemotherapy regimens and finally in prospective randomised trials.
